# Exploring Differential Perceptions of Artificial Intelligence in Health Care Among Younger Versus Older Canadians: Results From the 2021 Canadian Digital Health Survey

**DOI:** 10.2196/38169

**Published:** 2023-04-28

**Authors:** Karin Cinalioglu, Sasha Elbaz, Kerman Sekhon, Chien-Lin Su, Soham Rej, Harmehr Sekhon

**Affiliations:** 1 Department of Psychiatry Lady Davis Institute for Medical Research Jewish General Hospital Montreal, QC Canada; 2 Department of Psychiatry Faculty of Medicine and Health Sciences McGill University Montreal, QC Canada; 3 Department of Psychology Université du Québec à Montréal (UQAM) Montreal, QC Canada; 4 Temerty Faculty of Medicine University of Toronto Toronto, ON Canada; 5 Department of Psychiatry McLean Hospital Harvard Medical School Boston, MA United States

**Keywords:** artificial intelligence, telehealth, telemedicine, older adult, perception, technology, public opinion, national survey, Canada, Canadian, attitude, adoption, trust, satisfaction

## Abstract

**Background:**

The changing landscape of health care has led to the incorporation of powerful new technologies like artificial intelligence (AI) to assist with various services across a hospital. However, despite the potential outcomes that this tool may provide, little work has examined public opinion regarding their use.

**Objective:**

In this study, we aim to explore differences between younger versus older Canadians with regard to the level of comfort and perceptions around the adoption and use of AI in health care settings.

**Methods:**

Using data from the 2021 Canadian Digital Health Survey (n=12,052), items related to perceptions about the use of AI as well as previous experience and satisfaction with health care were identified. We conducted Mann-Whitney *U* tests to compare the level of comfort of younger versus older Canadians regarding the use of AI in health care for a variety of purposes. Multinomial logistic regression was used to predict the comfort ratings based on categorical indicators.

**Results:**

Younger Canadians had greater knowledge of AI, but older Canadians were more comfortable with AI applied to monitoring and predicting health conditions, decision support, diagnostic imaging, precision medicine, drug and vaccine development, disease monitoring at home, tracking epidemics, and optimizing workflow to save time. Additionally, for older respondents, higher satisfaction led to higher comfort ratings. Only 1 interaction effect was identified between previous experience, satisfaction, and comfort with AI for drug and vaccine development.

**Conclusions:**

Older Canadians may be more open to various applications of AI within health care than younger Canadians. High satisfaction may be a critical criterion for comfort with AI, especially for older Canadians. Additionally, in the case of drug and vaccine development, previous experience may be an important moderating factor. We conclude that gaining a greater understanding of the perceptions of all health care users is integral to the implementation and sustainability of new and cutting-edge technologies in health care settings.

## Introduction

### Background

Telemedicine or web-based health services and tools have been rapidly adopted into the health care system, in recent years, for a variety of applications [1-6]. One of the byproducts of the current medical technology revolution has been artificial intelligence (AI) [7], which is “a system’s ability to interpret external data correctly, to learn from such data, and to use those learnings to achieve specific goals and tasks through flexible adaptation” [8]. The use of AI is becoming pervasive in today’s day and age, with an annual rise of 40% and an estimated health care cost of US $150 billion by 2026 in the United States alone [9]. In 2018 and 2019, the American Medical Association and Canadian Medical Associations passed the first policy and directive, respectively, on AI applications in health care leading to its rise in recent years [10]. Moreover, this rapid increase in this new technology may prove beneficial for health systems, allowing for a more accessible, efficient, and inexpensive alternative to in-person patient monitoring. Further, its applications may be far-reaching such as with the keeping of medical records or remote delivery of care, treatment, and diagnosis applications to improve the quality of care patients are receiving.

Scientific curiosity regarding the potential affordances of AI in health care has grown considerably as well. For example, over the last 2 decades, more than 20,000 results of “AI in healthcare” can be found through a PubMed search. Recent work has documented the potential beneficial impacts of AI’s incorporation into health care such as helping with patient safety outcomes [11]. Moreover, there has been a nearly ubiquitous incorporation of AI in health care, with applications in disease monitoring, diagnosis, personalized treatment plans, treatment response, imaging, and data management, as well as in diverse fields such as neurology, cardiology, oncology, and psychiatry (eg, skin cancer screening, mental health, and Paro) [12-18]. Thus, it seems that AI may represent a flexible tool that can better aid health care professionals.

The sustainability of web-based care and AI-based technologies relies on numerous factors including the ease of use and perceptions of patient populations. Recent evidence shows that the widespread adoption of AI tools in health care by stakeholders is dependent on the positive perceptions and acceptability of AI among users [19-22]. Currently, literature is lacking on patient perceptions about AI in health care, particularly in the older adult population, with a large gap in our understanding of their views on AI [23-25]. Other eHealth technologies have been known to be accepted and used, prepandemic, in younger demographics [26].

With the current COVID-19 pandemic and evolving health care trends with increased uptake of digital health modalities among both older and younger Canadians alike, it has been noted that there is an improvement in opinions toward and overall acceptability of technology-assisted health practices [27,28]. To ensure proper adoption of particularly beneficial technologies that may greatly improve health-related services and subsequent outcomes, it is of interest to researchers to examine the opinions of the society in which they need to implement the tool.

Given the rapid acceleration in the incorporation of AI tools in health care settings, there is a need to evaluate the opinions and acceptability of AI in health care among patients. Furthermore, the current COVID-19 pandemic expediting the adoption of web-based care highlights the need to better understand whether past experience and satisfaction with web-based care help inform the perceptions of patients on the incorporation of AI within health care. As such, this study aims to describe the perceptions of older Canadians with regard to the use of AI within health care, as compared to younger Canadians, and examine whether this relationship is predicted by more prior experience and satisfaction with web-based care.

### Objectives and Hypotheses

Our primary objective in this study was to compare the perceptions of the use of AI (*primary outcome*) in health care among younger versus older Canadians. We theorize that younger individuals would have more favorable opinions and perceptions of the use of AI in health care as they tend to have more knowledge and firsthand experience using emerging technology compared to older Canadians. As a secondary objective, we aimed to explore the role of previous experience and satisfaction with web-based care in shaping more positive perceptions of the use of AI in health care. It was hypothesized that those survey respondents who had more prior experience and higher satisfaction rates with web-based health care would have more positive perceptions of the use of AI in health care.

## Methods

### Study Design and Survey

This study used an archival data analysis approach of a 2021 national survey (Canada Health Infoway, 2021) [29]. The Canadian Digital Health Survey is a national open data set comprising a series of surveys conducted annually commissioned by Canada Health Infoway (Infoway) through a third-party vendor. The survey questions address specific aspects of digital health awareness, usage, and perceptions among Canadians to provide actionable insights to help advance the digital health landscape in Canada. The data collection for the 2021 survey took place between July 14 and August 6, 2021, via computer-assisted web interviewing technology.

### Participants

The survey was conducted with 12,052 Canadians over the age of 16 years and it includes questions that will provide actionable insights to help advance the digital health landscape in Canada. The original data set had 5 age categories. To better address our objectives in this study, we regrouped all participants under 2 age categories: a younger (16-54 years old, 7644/12,052, 63%) and an older group (>54 years old, 4408/12,052, 37%).

### Data Selection and Analyses

To test our primary objective, items related to comfort and perceptions about the use of AI were identified. Selected items include comfort using personal data with informed consent (Q42), comfort using personal data without informed consent as long as the information is deidentified (Q43), comfort with the uses of AI within health care in monitoring and predicting health conditions (Q44r1), comfort with AI for decision support (44r2), comfort with AI for diagnostic imaging (44r3), comfort with AI for precision medicine (Q44r4), comfort with AI for drug and vaccine development (Q44r5), comfort with AI for disease monitoring at home (Q44r6), comfort with AI for tracking epidemics (Q44r7), and comfort with AI in optimizing workflow to save time for health care (q44r8). To address our secondary objective, 1 item relating to previous experience with digital services to help or support mental health issues (Q14_Lr12) and 1 item relating to satisfaction with virtual health care access (Q18r2) were identified. Our main independent outcome was perceptions of the use of AI in health care.

### Analyses Type

The Mann-Whitney *U* test, the nonparametric alternative to the parametric 2-sample t-test, was used to compare the level of comfort of younger versus older Canadians regarding the use of AI in health care for a variety of purposes. Additionally, given the categorical nature of the survey with multiple response levels to each question, a multinomial logistic regression was used to predict the categorical placement of individuals’ choices [30]. For the analysis, the “older” group as well as the “very uncomfortable” category served as references. Both SPSS (IBM Corp, 2020) and R version x64 4.0.3. (R Core Team, 2021) were used to analyze the data. The significance threshold was set to .05.

## Results

### Demographic Data

The large-scale data set captured the responses of 12,052 participants from across Canada, with ages ranging from 16 years to greater than 55 years. When asked how individuals described their gender identity, 5648 individuals identified themselves as male (47%), 6723 (52%) identified themselves as female, and 131 (1%) reported being of “other gender,” which included nonbinary, two-spirited, transgender, and so forth. The participants’ ages ranged as follows: 16 to 24 years old (1260/12,052, 10%); 25 to 34 years old (1843/12,052, 15%); 35 to 44 years old (2141/12,052, 18%); 45 to 54 years old (2400/12,052, 20%), and 55 years and older (4408/12,052, 37%). Respondents were primarily from Ontario (4844/12,052, 40%), Quebec (2867/12,052, 24%), the Canadian Prairies (2263/12,052, 19%), British Columbia/Northwest Territories (1330/12,052, 11%), and from parts of Atlantic Canada (748/12,052, 6%). The demographic data for the study can be found in Table 1.

**Table 1:** Demographic data (N=12,052).

Variables		Participants, n (%)
**Age (years)**		
	16-24	1260 (10)
	25-34	1843 (15)
	35-44	2141 (18)
	45-54	2400 (20)
	>55	4408 (37)
**Gender**		
	Male	5648 (47)
	Female	6273 (52)
	Other	131 (1)
**Location**		
	Ontario	4844 (40)
	Quebec	2867 (24)
	British Columbia/Northwest Territories	1330 (11)
	Atlantic Canada	748 (6)
	Canadian Prairies	2263 (19)
**Language spoken at home**		
	English	8981 (74)
	French	2551 (21)
	Chinese (Mandarin, Cantonese)	174 (1)
	Other	345 (3)
	Not discussed	1 (0)
**Health insurance coverage**		
	No coverage	1060 (9)
	I don’t know	574 (5)
	Prefer not to answer	280 (2)
	Public/provincial or Indigenous, First Nations, Inuit, and Métis (FNIM)	4065 (34)
	Private coverage paid by you or family	1903 (16)
	Private coverage paid by other	4170 (35)
**Employment status**		
	Full-time	5763 (48)
	Part-time	1282 (11)
	Homemaker	424 (3)
	Unemployed	572 (5)
	Retired	2619 (22)
	Disabled	348 (3)
	Student	788 (6)
	Other	140 (1)
	Prefer not to say	116 (1)
**Education**		
	High school (secondary school) diploma or equivalent	2571 (21)
	Registered apprenticeship or other trades certificate or diploma	643 (5)
	A college, CEGEP, or other nonuniversity certificate or diploma	2865 (24)
	A university certificate, or diploma or degree (eg, Bachelor’s degree)	4058 (34)
	Masters	1235 (10)
	PhD (or any equivalent doctoral degree)	199 (2)
	Medical or paramedical professional degree (eg, MD)	107 (1)
	Other—please specify	86 (1)
	None of the above	158 (1)
	Prefer not to answer	130 (1)

Following our age recoding of younger (ie, <55 year olds) and older (ie, >55 year olds) individuals, of the 12,052 participants, the majority (7644/12,052, 63%) were considered younger participants, whereas considerably fewer (4408/12,052, 37%) were considered older. A chi-square test revealed that the distribution of younger respondents from Ontario (n=3052), Quebec (n=1933), the Canadian Prairies (n=1399), British Columbia/Northwest Territories (n=826), and Atlantic Canada (n=434) was different from the distribution of older participants from Ontario (n=1792), Quebec (n=934), the Canadian Prairies (n=864), British Columbia/Northwest Territories (n=504), and Atlantic Canada (n=314; *χ*^2^_4_ N=12,052=33.040; *P*<.001). Several languages were also used in Canadian households. For example, 8987 (74%) of the respondents spoke English at home, 2551 (21%) spoke French, 174 (1%) spoke Chinese (Mandarin or Cantonese), and the remaining group spoke a different language or did not declare their spoken language at home.

### Objective 1: Perceptions of the Use of AI Among Older Versus Younger Canadians

**Figure 1:**
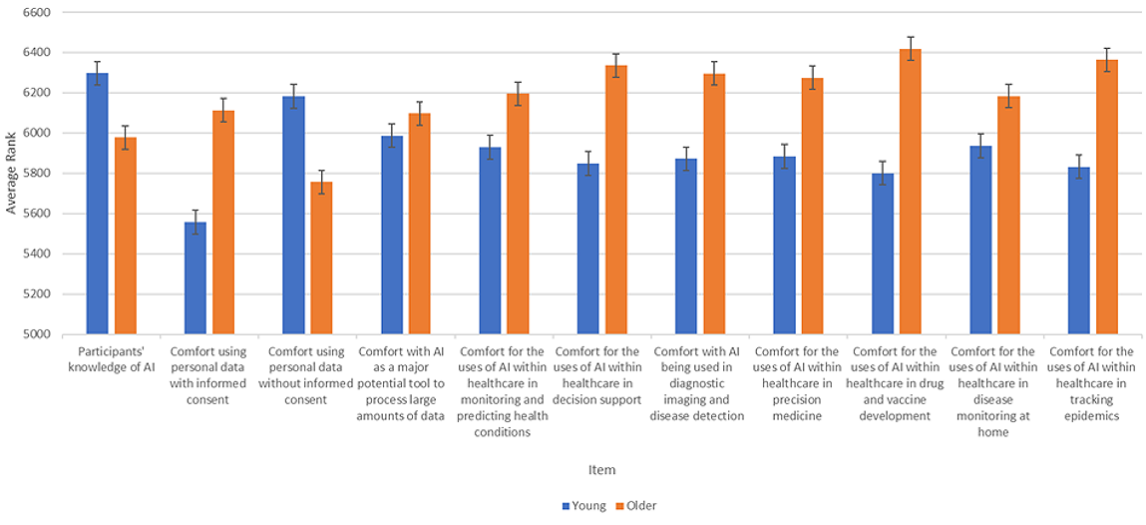
Differences between older compared to younger Canadians’ perceptions of artificial intelligence (AI). Error bars are represented by the standard error. The figure shows that older adults typically rate being more comfortable with AI in health care compared to younger individuals.

Table 2 reports the details of the Mann-Whitney *U* test of differences between perceptions of the use of AI among older versus younger Canadians. Additionally, Figure 1 presents a visualization of the differences for each question per group (younger versus older Canadians). By examining participants' knowledge of AI, we found that younger individuals (6296.97), defined as those under 55 years old, had significantly higher AI knowledge compared to older individuals (*P*<.001). Despite younger Canadians having comparatively higher knowledge, older Canadians were more comfortable with the use of their personal data with informed consent (*P*=.03) as well as the use of their personal data without informed consent (*P*<.001). This may represent a lack of understanding as to what type of information older Canadians are providing health providers for the sake of achieving healthier lives.

Regarding the possible affordances that AI can provide to health care and medical improvements, older Canadians reported higher ratings of comfort with AI in *monitoring and predicting health conditions* (*P*<.001), *decision support* (*P*<.001), *diagnostic imaging*, *precision medicine* (*P*<.001), *drug and vaccine development* (*P*<.001), *disease monitoring* at home (*P*<.001), and for *tracking epidemics* (*P*<.001). Overall, these findings suggest that older Canadians may see the strengths that AI can provide compared to younger Canadians.

Finally, considering AI-guided solutions applied to data management within health care, we found that older Canadians rated higher comfort with AI applied to *optimizing workflow* (*P*<.001), but found no significant differences between younger and older Canadians’ comfort ratings with AI as a *potential tool to process large amounts of data* (*P*=.08). These findings suggest that while older Canadians may see the potential speed improvement that AI can provide health care services as it may directly affect them (eg, speeding up intake processes), both groups may not find it particularly noteworthy when applied to less proximal matters such as data processing.

**Table 2:** Mann-Whitney U test of differences between younger versus older Canadians.

Item and group			Participants, n		Rank average		Mann Whitney *U*		*P* value
**Participants' knowledge of AI^a^**							14,779,930.50		<.001
	Younger	7644		6296.97					
	Older	4408		5557.48					
**Comfort using personal data with informed consent**							16,468,338.00		.30
	Younger	7644		5976.91					
	Older	4408		6112.49					
**Comfort using personal data without informed consent**							15,654,590.50		.001
	Younger	7644		6182.54					
	Older	4408		5755.90					
**Comfort with AI as a major potential tool to process large amounts of data**							16,540,240.00		.79
	Younger	7644		5986.32					
	Older	4408		6096.18					
**Comfort for the uses of AI within health care in monitoring and predicting health conditions**							16,104,735.50		<.001
	Younger	7644		5929.35					
	Older	4408		6194.98					
**Comfort for the uses of AI within health care in decision support**							15,483,997.00		<.001
	Younger	7644		5848.14					
	Older	4408		6335.80					
**Comfort with AI being used in diagnostic imaging and disease detection**							15,662,216.00		<.001
	Younger	7644		5871.46					
	Older	4408		6295.34					
**Comfort for the uses of AI within health care in precision medicine**							15,755,037.00		<.001
	Younger	7644		5883.60					
	Older	4408		6274.31					
**Comfort for the uses of AI within health care in drug and vaccine development**							15,122,200.00		<.001
	Younger	7644		5800.81					
	Older	4408		6417.87					
**Comfort for the uses of AI within health care in disease monitoring at home**							16,158,032.00		<.001
	Younger	7644		5936.32					
	Older	4408		6182.88					
**Comfort for the uses of AI within health care in tracking epidemics**							15,361,074.50		<.001
	Younger	7644		5832.06					
	Older	4408		6363.68					
**Comfort for the uses of AI within health care in optimizing workflow to save time for health care**							16,202,367.50		<.001
	Younger	7644		5942.12					
	Older	4408		6172.83					

^a^AI: artificial intelligence.

### Objective 2: Previous Experience and Satisfaction With Web-based Health Care

We present the summarized details of the fitted results for the multinomial regression model for all items in Multimedia Appendix 1. Both moderate and strong satisfaction ratings significantly predicted increases in comfort rating for older Canadians compared to younger Canadians in regard to using AI within health care in monitoring and predicting health conditions, using AI for decision support for diagnostic imaging and disease detection, precision medicine, drug and vaccine development, disease monitoring at home, tracking epidemics, and optimizing workflow to save time for health care*.* Examining the log odds suggests individuals who are more satisfied with these technologies will have a higher probability of being comfortable, notably in the case of older Canadians. Finally, we found a significant interaction between previous experience with AI, moderate satisfaction, and being very comfortable with the uses of AI within health care in drug and vaccine development. This suggests that positive previous experiences with AI can interact with satisfaction to result in being comfortable with their uses. In other words, previous experience with AI can positively *moderate* the relationship between satisfaction and comfort. Overall, these findings show that as satisfaction with AI increases so does comfort.

## Discussion

### Principal Findings

This study aimed to compare the perceptions of younger (<55 years old) and older (≥55 years old) Canadians on the uses and application of AI across various areas of health care with the overall goal of this work providing information to policy makers and health care professionals for guidance when implementing AI within health care. We hypothesized that younger individuals, who are generally assumed to be more exposed to and knowledgeable about emerging technology, would have more favorable opinions and responses to the use of AI in health care compared to older individuals. It was also hypothesized that having a previous experience with digital technologies that use AI as well as being *satisfied* with the reaction would predict more positive perceptions of Canadians.

Our results showed that despite being less knowledgeable about AI, older Canadians are significantly more comfortable with AI being applied in specific branches of health care than younger Canadians. These results also suggest that common assumptions about older groups’ difficulties with navigating technology, with lack of experience or knowledge of technology, and preference for traditional methods of care over web-based care may not be accurate. In fact, it may signal the changing tide of older generations of Canadians when it comes to new and emerging technologies. Older individuals are becoming increasingly familiar with and adept in their knowledge and use of technology. The digital divide is closing, with 73% of older populations using the internet in 2019 compared to 14% in 2000 [31]. Furthermore, the findings from Mitzner et al [26] suggest that once older individuals are able to perceive the benefits associated with certain technologies, they can perceive their use in a more favorable manner.

We did find that comfort with personal data without informed consent was significantly higher for younger responders than for older responders, however. One reason for this may be that older individuals may still experience a fear or concern regarding the technologies’ safety with regard to data privacy and confidentiality [32]. However, because individuals of today are more connected than ever given the COVID-19 repercussions worldwide, the emergent zeitgeist of what technology can afford may be seen more favorably. A study by Wang et al [33] provide further support for this finding, as participants in their investigation both had data privacy concerns but shared a willingness to contribute to further improvement of new and emerging technologies.

Examining the secondary hypothesis, we found that the interaction between previous experience and moderate satisfaction predicts increased comfort for the uses of AI within health care in drug and vaccine development. This can be indicative of the importance of positive experiences with the use of technology and web-based care in health care settings for patients. However, this interaction was not found for other perception items. Additionally, examining the findings, we see that younger people tend to have a lower probability of being moderately and very comfortable with the items relating to comfort for the uses of AI within health care in decision support and have a lower probability of being very comfortable for the item relating to comfort for the uses of AI within health care in tracking epidemics. One potential reason for this may be that younger people have less favorable opinions of health care and the health system overall. The findings from a study by Hargreaves et al [34] propose that overall younger people tend to have poorer experiences across all aspects of inpatient care (*P*<.001). In this study, we also found that people with previous experience tend to be more satisfied. Likewise, based on the coefficients of satisfaction, people with high satisfaction scores tend to be more comfortable with the use of AI in health care across different domains.

These findings lend support to Baltes’ [35] Selection Optimization Compensation theory, which proposes that as people get older, they allocate more resources toward loss management in an effort to attain their goals. Baltes [35] proposes that 3 fundamental life processes govern one’s life management: *selection*, which refers to one goal; *optimization* referring to the means one goes through to achieve their goals; and *compensation*, which refers to the individuals’ reactions to loss in their means to achieve their goals. Similar to previous work from Vaportzis et al, although in this study, goals and compensation were not measured, it can be seen that the older participants generally emphasized a greater interest in the various uses of AI in health care. One possibility may be that the participants would have a greater affinity for “optimizing” their behaviors [36].

### Limitations and Future Directions

There are a few limitations within this study. First, there may be an over-reliance on self-reported data and cross-sectional data. As such, we acknowledge that there may be a degree of self-report bias [37]. Additionally, given the nature of the study, only exploratory analyses were conducted. Furthermore, our study defined “older” adults as those 55 years of age and older. However, there is no general consensus on age ranges that clearly define at what point one is “older” as such decision-making based on these results should be carefully considered before action. Moreover, given the inclusion of comparatively younger individuals (ie, adults 55 years and older), this study may not align with other studies examining geriatrics and technology, some of which use the individual's chronological age of 65 years as a cut-off [38]. Finally, due to the nature of the data set, the psychometric properties of the questions cannot be determined, making it impossible to make any assertions regarding validity or reliability [39]. Nevertheless, given the exploratory nature of the survey questions coupled with the large, varied sample, the results provide insight into the nature of Canadians' views of AI in health care.

Given the relative lack of literature examining possible mediators of Canadians’ perceptions of AI, future work should aim to explore these possible variables. Additionally, AI becomes more ubiquitous in daily life [40] and within health care more specifically, longitudinal and qualitative investigations may be useful as they can provide more nuance into the changes (or stability) of Canadians’ views of and lived experiences of AI in their lives.

### Conclusions

Gaining a greater understanding of the perceptions of health care users is integral to the implementation and sustainability of new and cutting-edge technologies in health care settings. The results presented in this study, as well as other careful analyses and examinations of similar survey data may provide guidance in developing and implementing new technologies for various uses in the health care system. Furthermore, such data may be increasingly useful for policy makers and other professionals who work within health care and inform clinical practice and future research in this area. Future studies are needed to investigate the clinical impact of the use of AI for different patient populations.

## Appendix

Multimedia Appendix 1 Multinomial regression table for the level of comfort regarding various uses of AI in health care.

## Abbreviations

AI: artificial intelligence
