# Supplementary material for: Exploring Differential Perceptions of Artificial Intelligence in Health Care Among Younger Versus Older Canadians: Results From the 2021 Canadian Digital Health Survey
Source: J Med Internet Res. 2023 Apr 28;25:e38169. doi: 10.2196/38169 (PMC10182456; doi:10.2196/38169)
Supplement: Multimedia Appendix 1 [file jmir_v25i1e38169_app1.docx]

**Supplementary File**

*Multinomial regression table*

| Item | | Coef | SE | 95% CI [Lower to Upper] | Sig. |
| --- | --- | --- | --- | --- | --- |
| Comfort for the uses of AI within healthcare in monitoring and predicting health conditions | |  |  |  |  |
| Moderately uncomfortable | Intercept | .629 | 0.364 | [-0.085 to 1.343] | .084 |
|  | Younger | -.201 | 0.193 | [-0.581 to 0.178] | .298 |
|  | Previous experience | .044 | 0.812 | [-1.547 to 1.635] | .957 |
|  | Satisfaction-Moderately disagree | .010 | 0.442 | [-0.856 to 0.876] | .982 |
|  | Satisfaction-moderately agree | .718 | 0.384 | [-0.034 to 1.47] | .061 |
|  | Satisfaction-strongly agree | .101 | 0.389 | [-0.662 to 0.865] | .795 |
|  | Previous experience* satisfaction- Moderately disagree | -.377 | 1.019 | [-2.375 to 1.62] | .711 |
|  | Previous experience* satisfaction -moderately agree | .160 | 0.905 | [-1.614 to 1.934] | .860 |
|  | Previous experience* satisfaction -strongly agree | .260 | 0.929 | [-1.561 to 2.08] | .780 |
| Moderately comfortable | Intercept | 1.342 | 0.329 | [0.697 to 1.987] | 0 |
|  | Younger | -.292 | 0.172 | [-0.628 to 0.044] | .089 |
|  | Previous experience | .357 | 0.717 | [-1.048 to 1.761] | .619 |
|  | Satisfaction-Moderately disagree | .901 | 0.390 | [0.137 to 1.664] | .021** |
|  | Satisfaction-moderately agree | 1.364 | 0.348 | [0.682 to 2.046] | 0*** |
|  | Satisfaction-strongly agree | 1.019 | 0.349 | [0.336 to 1.702] | .003** |
|  | Previous experience* satisfaction- Moderately disagree | -.773 | 0.874 | [-2.485 to 0.939] | .376 |
|  | Previous experience* satisfaction -moderately agree | -.118 | 0.806 | [-1.699 to 1.462] | .883 |
|  | Previous experience* satisfaction -strongly agree | .070 | 0.817 | [-1.531 to 1.671] | .932 |
| Very comfortable | Intercept | 1.004 | 0.343 | [0.332 to 1.677] | .003 |
|  | Younger | -.270 | 0.175 | [-0.612 to 0.073] | .123 |
|  | Previous experience | .511 | 0.732 | [-0.925 to 1.946] | .486 |
|  | Satisfaction-Moderately disagree | .561 | 0.408 | [-0.238 to 1.36] | .168 |
|  | Satisfaction-moderately agree | 1.169 | 0.362 | [0.459 to 1.878] | .001** |
|  | Satisfaction-strongly agree | 1.269 | 0.361 | [0.561 to 1.977] | 0*** |
|  | Previous experience* satisfaction- Moderately disagree | -.959 | 0.908 | [-2.739 to 0.821] | .291 |
|  | Previous experience* satisfaction -moderately agree | -.458 | 0.826 | [-2.076 to 1.16] | .579 |
|  | Previous experience* satisfaction -strongly agree | .165 | 0.830 | [-1.461 to 1.791] | .842 |
| Comfort for the uses of AI within healthcare in decision support | |  |  |  |  |
| Moderately uncomfortable | Intercept | .204 | 0.307 | [-0.397 to 0.805] | .506 |
|  | Younger | .018 | 0.168 | [-0.312 to 0.348] | .914 |
|  | Previous experience | .880 | 0.730 | [-0.551 to 2.311] | .228 |
|  | Satisfaction-Moderately disagree | .547 | 0.372 | [-0.183 to 1.277] | .142 |
|  | Satisfaction-moderately agree | .758 | 0.322 | [0.126 to 1.389] | .019* |
|  | Satisfaction-strongly agree | .413 | 0.328 | [-0.230 to 1.056] | .208 |
|  | Previous experience* satisfaction- Moderately disagree | -.856 | 0.935 | [-2.689 to 0.977] | .360 |
|  | Previous experience* satisfaction -moderately agree | -.342 | 0.812 | [-1.934 to 1.249] | .673 |
|  | Previous experience* satisfaction -strongly agree | -.820 | 0.838 | [-2.462 to 0.822] | .328 |
| Moderately comfortable | Intercept | .768 | 0.281 | [0.216 to 1.319] | .006 |
|  | Younger | -.305 | 0.149 | [-0.598 to -0.013] | .041* |
|  | Previous experience | .456 | 0.731 | [-0.977 to 1.889] | .533 |
|  | Satisfaction-Moderately disagree | 1.178 | 0.340 | [0.512 to 1.844] | .001** |
|  | Satisfaction-moderately agree | 1.574 | 0.296 | [0.993 to 2.155] | 0*** |
|  | Satisfaction-strongly agree | 1.333 | 0.299 | [0.746 to 1.92] | 0*** |
|  | Previous experience* satisfaction- Moderately disagree | -.381 | 0.899 | [-2.143 to 1.381] | .672 |
|  | Previous experience* satisfaction -moderately agree | -.143 | 0.804 | [-1.718 to 1.432] | .859 |
|  | Previous experience* satisfaction -strongly agree | .099 | 0.811 | [-1.490 to 1.688] | .903 |
| Very comfortable | Intercept | .357 | 0.312 | [-0.255 to 0.969] | .253 |
|  | Younger | -.589 | 0.155 | [-0.894 to -0.285] | 0*** |
|  | Previous experience | 1.637 | 0.708 | [0.25 to 3.024] | .021* |
|  | Satisfaction-Moderately disagree | .924 | 0.377 | [0.186 to 1.662] | .014 |
|  | Satisfaction-moderately agree | 1.385 | 0.329 | [0.740 to 2.029] | 0*** |
|  | Satisfaction-strongly agree | 1.573 | 0.330 | [0.926 to 2.219] | 0*** |
|  | Previous experience* satisfaction- Moderately disagree | -1.617 | 0.913 | [-3.407 to 0.172] | .076 |
|  | Previous experience* satisfaction -moderately agree | -1.151 | 0.788 | [-2.696 to 0.394] | .144 |
|  | Previous experience* satisfaction -strongly agree | -.720 | 0.791 | [-2.270 to 0.829] | .362 |
| Comfort with AI being used in diagnostic imaging and disease detection | |  |  |  |  |
| Moderately uncomfortable | Intercept | .132 | 0.372 | [-0.597 to 0.861] | .722 |
|  | Younger | .222 | 0.201 | [-0.172 to 0.617] | .269 |
|  | Previous experience | .382 | 0.711 | [-1.011 to 1.775] | .591 |
|  | Satisfaction-Moderately disagree | .321 | 0.453 | [-0.567 to 1.208] | .479 |
|  | Satisfaction-moderately agree | .916 | 0.392 | [0.147 to 1.685] | .020* |
|  | Satisfaction-strongly agree | .346 | 0.402 | [-0.442 to 1.134] | .390 |
|  | Previous experience* satisfaction- Moderately disagree | -.669 | 0.961 | [-2.553 to 1.215] | .486 |
|  | Previous experience* satisfaction -moderately agree | -.622 | 0.797 | [-2.184 to 0.939] | .435 |
|  | Previous experience* satisfaction -strongly agree | -.134 | 0.844 | [-1.788 to 1.519] | .873 |
| Moderately comfortable | Intercept | 1.142 | 0.321 | [0.512 to 1.771] | 0 |
|  | Younger | -.087 | 0.176 | [-0.431 to 0.257] | .621 |
|  | Previous experience | .105 | 0.652 | [-1.173 to 1.382] | .872 |
|  | Satisfaction-Moderately disagree | .929 | 0.390 | [0.165 to 1.693] | .017** |
|  | Satisfaction-moderately agree | 1.502 | 0.343 | [0.831 to 2.174] | 0*** |
|  | Satisfaction-strongly agree | 1.139 | 0.346 | [0.46 to 1.818] | .001** |
|  | Previous experience* satisfaction- Moderately disagree | -.463 | 0.847 | [-2.123 to 1.198] | .585 |
|  | Previous experience* satisfaction -moderately agree | -.416 | 0.727 | [-1.842 to 1.01] | .567 |
|  | Previous experience* satisfaction -strongly agree | .193 | 0.763 | [-1.303 to 1.688] | .800 |
| Very comfortable | Intercept | 1.040 | 0.330 | [0.394 to 1.686] | .002** |
|  | Younger | -.328 | 0.176 | [-0.674 to 0.017] | .063 |
|  | Previous experience | .125 | 0.674 | [-1.196 to 1.446] | .853 |
|  | Satisfaction-Moderately disagree | .945 | 0.399 | [0.162 to 1.728] | .018* |
|  | Satisfaction-moderately agree | 1.381 | 0.352 | [0.691 to 2.071] | 0*** |
|  | Satisfaction-strongly agree | 1.602 | 0.354 | [0.908 to 2.296] | 0*** |
|  | Previous experience* satisfaction- Moderately disagree | -.541 | 0.874 | [-2.254 to 1.171] | .536 |
|  | Previous experience* satisfaction -moderately agree | -.539 | 0.752 | [-2.012 to 0.933] | .473 |
|  | Previous experience* satisfaction -strongly agree | .258 | 0.780 | [-1.271 to 1.787] | .741 |
| Comfort for the uses of AI within healthcare in precision medicine | |  |  |  |  |
| Moderately uncomfortable | Intercept | .217 | 0.326 | [-0.422 to 0.856] | .506 |
|  | Younger | .057 | 0.173 | [-0.281 to 0.396] | .740 |
|  | Previous experience | .248 | 0.797 | [-1.314 to 1.81] | .756 |
|  | Satisfaction-Moderately disagree | .623 | 0.387 | [-0.135 to 1.382] | .107 |
|  | Satisfaction-moderately agree | .766 | 0.344 | [0.092 to 1.44] | .026* |
|  | Satisfaction-strongly agree | .477 | 0.352 | [-0.213 to 1.167] | .175 |
|  | Previous experience* satisfaction- Moderately disagree | .336 | 1.046 | [-1.714 to 2.385] | .748 |
|  | Previous experience* satisfaction -moderately agree | .086 | 0.875 | [-1.629 to 1.801] | .921 |
|  | Previous experience* satisfaction -strongly agree | -.191 | 0.905 | [-1.964 to 1.582] | .833 |
| Moderately comfortable | Intercept | .887 | 0.291 | [0.316 to 1.458] | .002 |
|  | Younger | -.083 | 0.154 | [-0.385 to 0.219] | .591 |
|  | Previous experience | .645 | 0.701 | [-0.730 to 2.02] | .358 |
|  | Satisfaction-Moderately disagree | .844 | 0.349 | [0.16 to 1.528] | .016** |
|  | Satisfaction-moderately agree | 1.350 | 0.308 | [0.746 to 1.954] | 0*** |
|  | Satisfaction-strongly agree | 1.182 | 0.314 | [0.568 to 1.797] | 0*** |
|  | Previous experience* satisfaction- Moderately disagree | .052 | 0.947 | [-1.804 to 1.908] | .956 |
|  | Previous experience* satisfaction -moderately agree | -.378 | 0.777 | [-1.901 to 1.146] | .627 |
|  | Previous experience* satisfaction -strongly agree | -.161 | 0.793 | [-1.715 to 1.394] | .839 |
| Very comfortable | Intercept | .321 | 0.324 | [-0.314 to 0.956] | .321 |
|  | Younger | -.163 | 0.160 | [-0.478 to 0.151] | .307 |
|  | Previous experience | 1.106 | 0.725 | [-0.316 to 2.527] | .127 |
|  | Satisfaction-Moderately disagree | .683 | 0.386 | [-0.074 to 1.44] | .077 |
|  | Satisfaction-moderately agree | 1.249 | 0.341 | [0.58 to 1.918] | 0*** |
|  | Satisfaction-strongly agree | 1.569 | 0.344 | [0.894 to 2.244] | 0*** |
|  | Previous experience* satisfaction- Moderately disagree | -.788 | 1.004 | [-2.755 to 1.179] | .432 |
|  | Previous experience* satisfaction -moderately agree | -.903 | 0.806 | [-2.483 to 0.676] | .262 |
|  | Previous experience* satisfaction -strongly agree | -.359 | 0.814 | [-1.955 to 1.237] | .659 |
| Comfort for the uses of AI within healthcare in drug and vaccine development | |  |  |  |  |
| Moderately uncomfortable | Intercept | -.214 | 0.373 | [-0.945 to 0.516] | .565 |
|  | Younger | .276 | 0.181 | [-0.078 to 0.631] | .126 |
|  | Previous experience | .835 | 0.78 | [-0.694 to 2.363] | .285 |
|  | Satisfaction-Moderately disagree | .890 | 0.445 | [0.019 to 1.762] | .045* |
|  | Satisfaction-moderately agree | .770 | 0.387 | [0.011-1.528] | .047* |
|  | Satisfaction-strongly agree | .392 | 0.392 | [-0.377 to 1.161] | .318 |
|  | Previous experience* satisfaction- Moderately disagree | .067 | 1.122 | [-2.131 to 2.265] | .952 |
|  | Previous experience* satisfaction -moderately agree | -1.199 | 0.840 | [-2.845 to 0.447] | .153 |
|  | Previous experience* satisfaction -strongly agree | -.644 | 0.871 | [-2.352 to 1.063] | .460 |
| Moderately comfortable | Intercept | 1.131 | 0.299 | [0.544 to 1.718] | 0 |
|  | Younger | -.107 | 0.153 | [-0.407 to 0.192] | .482 |
|  | Previous experience | .253 | 0.714 | [-1.147 to 1.653] | .723 |
|  | Satisfaction-Moderately disagree | .856 | 0.372 | [0.127 to 1.586] | .021* |
|  | Satisfaction-moderately agree | 1.061 | 0.315 | [0.443 to 1.678] | .001** |
|  | Satisfaction-strongly agree | .690 | 0.317 | [0.068 to 1.312] | .030* |
|  | Previous experience* satisfaction- Moderately disagree | .442 | 1.049 | [-1.614 to 2.498] | .673 |
|  | Previous experience* satisfaction -moderately agree | -.640 | 0.761 | [-2.133 to 0.852] | .400 |
|  | Previous experience* satisfaction -strongly agree | .116 | 0.786 | [-1.426 to 1.657] | .883 |
| Very comfortable | Intercept | .792 | 0.321 | [0.163 to 1.421] | .014 |
|  | Younger | -.435 | 0.154 | [-0.736 to -0.134] | .005** |
|  | Previous experience | 1.080 | 0.711 | [-0.313 to 2.474] | .129 |
|  | Satisfaction-Moderately disagree | 1.105 | 0.393 | [0.335 to 1.875] | .005** |
|  | Satisfaction-moderately agree | 1.286 | 0.337 | [0.625 to 1.946] | 0*** |
|  | Satisfaction-strongly agree | 1.342 | 0.338 | [0.68 to 2.004] | 0*** |
|  | Previous experience* satisfaction- Moderately disagree | -.724 | 1.064 | [-2.81 to 1.362] | .496 |
|  | Previous experience* satisfaction -moderately agree | -1.533 | 0.762 | [-3.026 to -0.04] | .044* |
|  | Previous experience* satisfaction -strongly agree | -.593 | 0.782 | [-2.126 to 0.94] | .449 |
| Comfort for the uses of AI within healthcare in disease monitoring at home | |  |  |  |  |
| Moderately uncomfortable | Intercept | .374 | 0.339 | [-0.291 to 1.039] | .270 |
|  | Younger | -.021 | 0.179 | [-0.371 to 0.329] | .906 |
|  | Previous experience | .048 | 0.725 | [-1.372 to 1.468] | .947 |
|  | Satisfaction-Moderately disagree | .658 | 0.410 | [-0.145 to 1.461] | .108 |
|  | Satisfaction-moderately agree | .592 | 0.353 | [-0.101 to 1.284] | .094 |
|  | Satisfaction-strongly agree | .459 | 0.364 | [-0.253 to 1.172] | .207 |
|  | Previous experience* satisfaction- Moderately disagree | -.253 | 0.973 | [-2.16 to 1.653] | .794 |
|  | Previous experience* satisfaction -moderately agree | .507 | 0.833 | [-1.126 to 2.14] | .543 |
|  | Previous experience* satisfaction -strongly agree | .235 | 0.846 | [-1.423 to 1.893] | .781 |
| Moderately comfortable | Intercept | .915 | 0.312 | [0.303 to 1.526] | .003** |
|  | Younger | -.204 | 0.160 | [-0.517 to 0.109] | .201 |
|  | Previous experience | .567 | 0.64 | [-0.687 to 1.82] | .376 |
|  | Satisfaction-Moderately disagree | 1.177 | 0.377 | [0.438 to 1.917] | .002** |
|  | Satisfaction-moderately agree | 1.450 | 0.325 | [0.813 to 2.086] | 0*** |
|  | Satisfaction-strongly agree | 1.349 | 0.333 | [0.697 to 2.001] | 0*** |
|  | Previous experience* satisfaction- Moderately disagree | -.458 | 0.861 | [-2.146 to 1.23] | .595 |
|  | Previous experience* satisfaction -moderately agree | .081 | 0.745 | [-1.379 to 1.541] | .913 |
|  | Previous experience* satisfaction -strongly agree | -.126 | 0.750 | [-1.595 to 1.344] | .867 |
| Very comfortable | Intercept | .709 | 0.323 | [0.075 to 1.342] | .028** |
|  | Younger | -.226 | 0.165 | [-0.549 to 0.098] | .172 |
|  | Previous experience | .383 | 0.670 | [-0.931 to 1.697] | .567 |
|  | Satisfaction-Moderately disagree | .616 | 0.395 | [-0.159 to 1.391] | .119 |
|  | Satisfaction-moderately agree | .861 | 0.338 | [0.199 to 1.522] | .011** |
|  | Satisfaction-strongly agree | 1.398 | 0.343 | [0.725 to 2.071] | 0*** |
|  | Previous experience* satisfaction- Moderately disagree | -.361 | 0.913 | [-2.151 to 1.428] | .692 |
|  | Previous experience* satisfaction -moderately agree | .292 | 0.778 | [-1.233 to 1.816] | .708 |
|  | Previous experience* satisfaction -strongly agree | .370 | 0.776 | [-1.15 to 1.891] | .633 |
| Comfort for the uses of AI within healthcare in tracking epidemics | |  |  |  |  |
| Moderately uncomfortable | Intercept | -.456 | 0.417 | [-1.274 to 0.362] | .274 |
|  | Younger | .265 | 0.235 | [-0.195 to 0.725] | .260 |
|  | Previous experience | -.266 | 0.834 | [-1.9 to 1.367] | .749 |
|  | Satisfaction-Moderately disagree | 1.021 | 0.514 | [0.014 to 2.028] | .047** |
|  | Satisfaction-moderately agree | .984 | 0.438 | [0.125 to 1.843] | .025** |
|  | Satisfaction-strongly agree | .581 | 0.450 | [-0.302 to 1.464] | .197 |
|  | Previous experience* satisfaction- Moderately disagree | .051 | 1.091 | [-2.087 to 2.19] | .962 |
|  | Previous experience* satisfaction -moderately agree | .558 | 0.975 | [-1.353 to 2.47] | .567 |
|  | Previous experience* satisfaction -strongly agree | .607 | 1.018 | [-1.388 to 2.602] | .551 |
| Moderately comfortable | Intercept | .815 | 0.327 | [0.175 to 1.456] | .013 |
|  | Younger | -.053 | 0.193 | [-0.43 to 0.325] | .785 |
|  | Previous experience | .324 | 0.605 | [-0.862 to 1.51] | .592 |
|  | Satisfaction-Moderately disagree | 1.380 | 0.420 | [0.556 to 2.204] | .001** |
|  | Satisfaction-moderately agree | 1.648 | 0.349 | [0.964 to 2.332] | 0*** |
|  | Satisfaction-strongly agree | 1.294 | 0.355 | [0.599 to 1.99] | 0*** |
|  | Previous experience* satisfaction- Moderately disagree | -.774 | 0.860 | [-2.459 to 0.911] | .368 |
|  | Previous experience* satisfaction -moderately agree | .173 | 0.751 | [-1.299 to 1.645] | .818 |
|  | Previous experience* satisfaction -strongly agree | .467 | 0.777 | [-1.056 to 1.99] | .548 |
| Very comfortable | Intercept | 1.421 | 0.309 | [0.816 to 2.026] | 0 |
|  | Younger | -.377 | 0.189 | [-0.747 to -0.007] | .046* |
|  | Previous experience | -.447 | 0.624 | [-1.67 to 0.777] | .474 |
|  | Satisfaction-Moderately disagree | 1.244 | 0.405 | [0.450 to 2.038] | .002** |
|  | Satisfaction-moderately agree | 1.552 | 0.333 | [0.9 to 2.204] | 0*** |
|  | Satisfaction-strongly agree | 1.634 | 0.337 | [0.974 to 2.295] | 0*** |
|  | Previous experience* satisfaction- Moderately disagree | -.108 | 0.869 | [-1.812 to 1.596] | .901 |
|  | Previous experience* satisfaction -moderately agree | .697 | 0.766 | [-0.805 to 2.199] | .363 |
|  | Previous experience* satisfaction -strongly agree | 1.074 | 0.788 | [-0.471 to 2.619] | .173 |
| Comfort for the uses of AI within healthcare in optimizing workflow to save time for healthcare | |  |  |  |  |
| Moderately uncomfortable | Intercept | -.405 | 0.424 | [-1.236 to 0.425] | .339 |
|  | Younger | 0 | 0.214 | [-0.42 to 0.42] | 1 |
|  | Previous experience | .965 | 0.751 | [-0.506 to 2.436] | .199 |
|  | Satisfaction-Moderately disagree | 1.281 | 0.511 | [0.279 to 2.283] | .012* |
|  | Satisfaction-moderately agree | 1.496 | 0.446 | [0.622 to 2.37] | .001** |
|  | Satisfaction-strongly agree | .946 | 0.449 | [0.067 to 1.825] | .035* |
|  | Previous experience* satisfaction- Moderately disagree | -.454 | 1.131 | [-2.671 to 1.763] | .688 |
|  | Previous experience* satisfaction -moderately agree | -.609 | 0.864 | [-2.301 to 1.084] | .481 |
|  | Previous experience* satisfaction -strongly agree | -.870 | 0.874 | [-2.583 to 0.843] | .320 |
| Moderately comfortable | Intercept | 1.132 | 0.316 | [0.512 to 1.752] | 0 |
|  | Younger | -.202 | 0.186 | [-0.566 to 0.162] | .277 |
|  | Previous experience | .042 | 0.659 | [-1.251 to 1.334] | .950 |
|  | Satisfaction-Moderately disagree | 1.363 | 0.404 | [0.57 to 2.156] | .001** |
|  | Satisfaction-moderately agree | 1.767 | 0.341 | [1.099 to 2.434] | 0*** |
|  | Satisfaction-strongly agree | 1.191 | 0.339 | [0.526 to 1.855] | 0*** |
|  | Previous experience* satisfaction- Moderately disagree | .143 | 1.022 | [-1.86 to 2.147] | 0.888 |
|  | Previous experience* satisfaction -moderately agree | -.087 | 0.769 | [-1.593 to 1.419] | .910 |
|  | Previous experience* satisfaction -strongly agree | .202 | 0.760 | [-1.287 to 1.691] | .791 |
| Very comfortable | Intercept | 1.166 | 0.316 | [0.546 to 1.786] | 0*** |
|  | Younger | -.274 | 0.186 | [-0.639 to 0.092] | .142 |
|  | Previous experience | .151 | 0.654 | [-1.13 to 1.433] | .817 |
|  | Satisfaction-Moderately disagree | .944 | 0.409 | [0.143 to 1.744] | .021** |
|  | Satisfaction-moderately agree | 1.384 | 0.342 | [0.714 to 2.054] | 0*** |
|  | Satisfaction-strongly agree | 1.422 | 0.338 | [0.759 to 2.085] | 0*** |
|  | Previous experience* satisfaction- Moderately disagree | .431 | 1.021 | [-1.57 to 2.433] | .673 |
|  | Previous experience* satisfaction -moderately agree | -.086 | 0.766 | [-1.588 to 1.415] | .911 |
|  | Previous experience* satisfaction -strongly agree | .110 | 0.753 | [-1.366 to 1.586] | .884 |

* *P* < .05

** *P* <.01

*** *P* < .001
